# Community-based models of care for management of type 2 diabetes mellitus among non-pregnant adults in sub-Saharan Africa: a scoping review protocol

**DOI:** 10.12688/f1000research.52114.1

**Published:** 2021-07-05

**Authors:** Emmanuel Firima, Lucia Gonzalez, Jacqueline Huber, Jennifer M. Belus, Fabian Raeber, Ravi Gupta, Joalane Mokhohlane, Madavida Mphunyane, Alain Amstutz, Niklaus Daniel Labhardt

**Affiliations:** 1 Swiss Tropical and Public Health Institute, Basel, Basel-Stadt, Switzerland; 2 University of Basel, Basel, Switzerland; 3 Solidarmed, Partnerships for Health, Butha-Buthe, Lesotho; 4 Non-communicable diseases department, Ministry of Health, Maseru, Lesotho; 5 Department of Infectious Diseases and Hospital Epidemiology, University Hospital Basel, Basel, Switzerland

**Keywords:** community-based care, diabetes mellitus, treatment outcome, engagement in chronic care, access to healthcare, sub-Saharan Africa

## Abstract

**Background:** The burden of type 2 diabetes mellitus (T2DM) is increasing in low- and middle-income countries, including sub-Sahara Africa (SSA). However, awareness of and access to T2DM diagnosis and care remain low in SSA, leading to delayed treatment, early morbidity, and mortality. Particularly in rural settings with long distances to health care facilities, community-based care models may contribute to increased timely diagnosis and care. This scoping review aims to summarize and categorize existing models of community-based care for T2DM among non-pregnant adults in SSA, and to synthesize the evidence on acceptance, clinical outcomes, and engagement in care.

**Method and analysis:** This review will follow the framework suggested by Arskey and O’Malley, which has been further refined by Levac *et al.* and the Joanna Briggs Institute. Electronic searches will be performed in Medline, Embase, Cumulative Index to Nursing and Allied Health Literature (CINAHL) and Scopus, supplemented with backward and forward citation searches. We will include cohort studies, randomized trials and case-control studies that report cases of non-pregnant individuals diagnosed with T2DM in SSA who receive a substantial part of care in the community. Our outcomes of interest will be model acceptability, blood sugar control, end organ damage, and patient engagement in care. A narrative analysis will be conducted, and comparisons made between community-based and facility-based models, where within-study comparison is reported.

**Conclusion:** Care for T2DM has become a global health priority. Community-based care may be an important add-on approach especially in populations with poor access to health care facilities. This review will inform policy makers and program implementers on different community-based models for care of T2DM in SSA, and critically appraise their acceptability and clinical outcomes. It will further identify evidence gaps and future research priorities in community-based T2DM care.

## Introduction

Globally, there are about 463 million people living with diabetes mellitus, representing 9.3% of the global population aged 20 – 79 years. ^1^ This number is projected to rise to 700 million people in 2045. ^1^ Approximately 95% of diabetes mellitus cases are due to type 2 diabetes mellitus (T2DM), characterized by chronic hyperglycemia resulting from a decrease in insulin secretion, or insulin resistance. ^2^^,^^3^ The chronic hyperglycemia results in a wide range of long-term complications such as atherosclerosis, coronary heart disease, peripheral neuropathy, diabetic foot syndrome, renal disease and retinopathy. ^2^ The burden of diabetes mellitus disproportionately affects low- and middle-income countries. Of the 700 million projected cases by 2045, low- and middle-income countries will account for an estimated 630 million. ^4^ In sub-Saharan Africa (SSA), 20 million people currently live with diabetes with a projected increase to 47 million people by 2045. ^5^

It has been reported that in SSA only 50% of persons with T2DM are aware of their diagnosis ^5^ and only 29% of those are engaged in diabetes care. ^6^ Late diagnosis and poor treatment contribute to high rates of T2DM complications in the region, ^7^ with rising cases of retinopathy, nephropathy, and cardiomyopathy. ^7^ As mortality and morbidity due to T2DM are expected to grow substantially in the region, a widely variegated approach to diagnosis and care is essential to increase awareness and treatment coverage. Such approaches should take into account the economic, geographical and socio-cultural characteristics, and the needs of the population. ^8^

Community-based healthcare utilizes the various supportive structures in the community such as family, peers, lay health workers, outreach health posts, community-based- and faith-based organizations, to deliver convenient, affordable, and effective care. As part of an integrated health system, community-based care emphasizes the localization of care close to the patient’s residence rather than in a hospital or clinic. ^9^ The advantages of this approach include community ownership of health responsibility, identification and treatment of diseases at an early stage which reduces health costs faced by the patient and the health system. ^9^ Task-shifting from physicians to nurses or lay cadres is an essential component of community-based care. ^10^ In the HIV/AIDS response, task-shifting and community care have yielded positive results, improving linkage to care, engagement in care, and patient clinical outcomes. ^11^^–^^14^ T2DM programs could leverage on the lessons learnt and the success of this approach to improve screening and early diagnosis, as well as engagement in care. Currently, however, there is little evidence about T2DM community-based care models in SSA and how they perform with regards to acceptance, clinical endpoints, and long-term patient engagement in care.

## Study rationale

To inform future policies and programs for T2DM in SSA, this scoping review aims to summarize and categorize models of T2DM community-based care among non-pregnant adults in SSA, and to synthesize evidence on acceptance, clinical outcomes, and patient engagement in care. It will further identify evidence gaps and future research priorities in community-based T2DM care.

## Method and analysis

### Study design

We decided to use the scoping review approach to identify community-based models of T2DM care in SSA as the approach is well-suited to produce an overview of research evidence within the subject area, and on this particular topic. Using this approach, we will not conduct quality appraisal of selected studies, as we anticipate heterogeneity in the studies in terms of design and methodology. However, the scoping review approach will enable us to compile, categorize, and describe the existing evidence and its capacity to contribute to acceptable and quality T2DM care, which will inform practice, policy-making and future research.

We will conduct this scoping review using the six-stage approach initially developed by Arskey and O’Malley, which has been further refined by Levac *et al.* (2010) and the Joanna Briggs Institute methods of evidence synthesis, to ensure efficiency, quality, and reproducibility, as well as allow for critical appraisal of the findings. ^15^^–^^17^ This approach recommends the following stages:

1. identifying the research question;
2. identifying relevant studies;
3. selecting studies;
4. charting the data;
5. collating, summarising and reporting the results;
6. expert consultation (optional and included).

### Stage 1: Identifying the research questions

An iterative process guided by the PICO framework ( Table 1) was undertaken to identify the research questions, following consultations with experts as well as within our longstanding research teams in Switzerland and Lesotho. During this process we realized that we would need to include studies that assess community-based T2DM care models on their own as well as studies that compare community-based T2DM care models versus facility-based models. Thus, question 3 below will only be answered by studies including a comparison. Following this process, three research questions were identified:

1. What kind of community-based T2DM care models among non-pregnant adults exist in SSA?
2. What are clinical outcomes of community-based T2DM care models in SSA in terms of acceptability to both patient and care provider, blood sugar control, end organ damage, and patient engagement in care?
3. How do community-based T2DM care models in SSA perform compared to facility-based care models (within study comparison)?

**Table 1.** The PICO framework.

Criteria	Determinants
Population	Adult persons with non-gestational type 2 diabetes mellitus in sub-Saharan Africa
Intervention	Community-based care delivery
Comparison	Facility-based care (where available)
Outcome	Acceptability, Fasting blood glucose, Random blood glucose, glycated haemoglobin (HbA1c), engagement in care, development of T2DM-related complications

### Stage 2: Identifying relevant studies - search terms and inclusion/exclusion criteria Search strategy

We will conduct searches in Medline, Embase, Cumulative Index to Nursing and Allied Health Literature (CINAHL) and Scopus. The initial search will be developed for Embase (Elsevier). The search string is divided into three parts, namely “community-based care”, “type 2 diabetes” and “sub-Saharan Africa”. The search strategy will include identification of Emtree terms and keywords relating to each part of the search string. The research team will develop the search string iteratively, based on preliminary searches.

**Figure 1.**
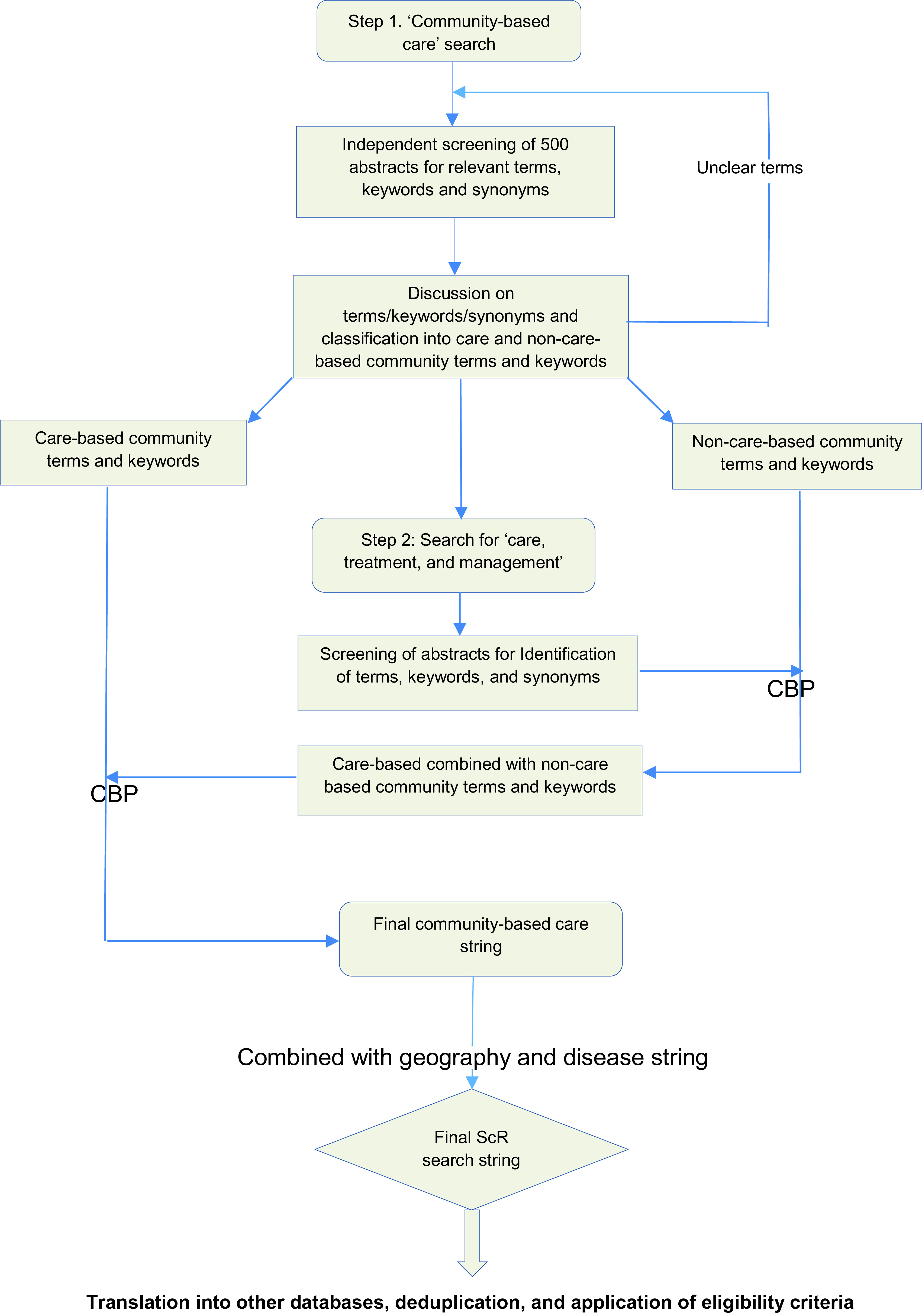
Flow diagram of search and study selection process. CBP = combined with Boolean and proximity operators. ScR = scoping review.

In an initial step, search will be conducted for the concept ‘community-based care’, to identify different terms and keywords used in the literature to describe such out-of-facility care. The first 500 abstracts will be screened by the reviewers (EF, LG, JB, JH, FR) to also identify relevant synonyms. Terms and keywords will be considered ‘care-based community terms and keywords’ if they describe a care, treatment, or management-centred activity outside of a traditional facility setting. Terms and keywords will be considered ‘non-care-based community terms and keywords’ if they only describe activity outside of traditional facility setting without a care, treatment, or management-centred component. In a following step, search will be conducted for the concept ‘care, treatment, or management’. Similarly, the abstracts will be screened for relevant terms and keywords, which will then be combined with the non-care-based community terms and keywords using Boolean and proximity operators; the latter combination will be associated with the care-based community terms and keywords for a final search string for the concept ‘community-based care’; See Figure 1.

During the preliminary search phase, the research team observed that some authors combined the reporting of diabetes mellitus, arterial hypertension or other cardiovascular conditions. Thus, the search string for the disease concept ‘type 2 diabetes mellitus’ will also include terms for hypertension and cardiovascular diseases. The string related to the geographical concept will be developed based on Campbell *et al.* ^19^ and the United Nations standard country or area codes for statistical use. ^20^

Following development of search strings for each concept, the search will be carried out in a stepwise, building block fashion which will be connected to obtain a final total of relevant publications in the database. The search string will then be translated into other databases using Polyglot Search Translator (Systematic Review Accelerator). ^18^ The design of the search strategy will be conducted in consultation with a medical librarian. Details of the search including a preliminary search string are available as *extended data* on Figshare. ^21^ Language restrictions will not be placed on retrieved studies. Date restrictions will also not be placed on reviewed articles. From articles extracted for full text screening, a forward and backward search will be conducted for relevant references in the selected articles as well as for articles that cite the selected studies.

### Criteria for identification of studies included in this review

***Studies***

We will include primary studies that have examined community-based models of care among patients with T2DM. Systematic or other reviews on community-based models of care will be included as a source of relevant original publications.

***Participants***

We will include studies that involved adults who have been diagnosed with T2DM using the standard diagnostic criteria. These adults will be resident and receiving care for their condition in sub-Saharan Africa.

***Intervention***

Intervention will be delivery of care different from the traditional facility-based care model, which attempts to make care available in the community, at patients’ homes or a central, non-formal health facility location where patients with similar conditions can access care. See Table 2 for components of a community-based model of care.

**Table 2.** Components of a community-based model of care.

**WHO**	• Any professional and non-professional cadre • Doctors, medical non-physician clinicians,nurses, pharmacists, community health workers (and similar), peers, self-care, psychologists and social workers, family members • Traditional healers (community members not providing western health care approaches • If non-professional providers: whether the project provides (or not) supervision and training from medical providers (inclusion criteria).
**POPULATION**	• Individuals who screen positive for type 2 diabetes mellitus.
**WHERE**	• Outside of the compound of a permanent health care facility. This may include, but not restricted to: community-based settings: outreach services, home-based care, places used for gathering (religious centres, schools, markets, shops) or delivering other services to citizens. Also, it includes e-health interventions.
**HOW OFTEN**	• Model foresees a reduction in number of patients visits to the permanent health facility, as compared to the standard of care. • The community part should not be an add-on to the care at the facility, but substitute some of the patient’s contact with facilities.
**WHAT**	Treatment provision in the community should include one of the following components: • Long-term medication prescription/distribution • Point of care monitoring (e.g. with glucometer) • Long-term lifestyle change support (at least 1 follow up encounter with a care provider) The following elements may be part of the model and will be described: • Diagnosis of chronic complications • Pharmaceutic treatment • Screening and early diagnosis of disease • Rehabilitation • Behavioural interventions, health promotion, education

***Comparator***

With facility-based care, where available.

***Outcome***

Of primary interest will be clinical outcomes like blood glucose indices and T2DM complications. Also of interest will be engagement in care, and acceptability of care to patients and providers.

See Table 3 for details.

**Table 3.** Inclusion/exclusion criteria

Parameter	Inclusion criteria	Exclusion criteria
Population	• Individuals aged 18 years and above, all genders, ethnic groups, education levels, socio-economic levels • Diagnosed with type 2 diabetes mellitus (T2DM) using the standard diagnostic criteria • In any of Angola, Benin, Botswana, Burkina Faso, Burundi, Cameroon, Central African Republic, Chad, Congo, Cote d'Ivoire, Equatorial New Guinea, Eritrea, Ethiopia, eSwatini, Gabon, Gambia, Ghana, Guinea, Guinea-Bissau, Kenya, Lesotho, Liberia, Madagascar, Malawi, Mali, Mauritania, Mauritius, Mozambique, Namibia, Niger, Nigeria, Rwanda, Senegal, Sierra Leone, Somalia, South Africa, Sudan (North, South), United Republic of Tanzania, Togo, Uganda, Zaire, Zambia, Zimbabwe.	Individuals diagnosed as having impaired glucose tolerance, pregnant women
Intervention	Community-based care, that is a form of patient care differing from the traditional facility-based model considering the location, frequency of contact with care provider and cadre of staff (see Table 2)	
Comparator	Traditional facility-based care, where available.	
Outcomes	Studies reporting at least one the following outcomes will be included: • Clinical outcomes: of interest are tasting blood glucose, random blood glucose, glycated haemoglobin (HbA1c), episodes of hypoglycaemia and hyperglycaemia, adherence to T2DM medication, development of complications like retinopathy, nephropathy, diabetic foot syndrome, cardiovascular diseases and cerebrovascular diseases • Engagement in care • Acceptability to patients or providers	Studies not reporting any of the outcomes
Study design	• Prospective or retrospective cohorts • Randomised control trials • Non-randomised control trials • Quasi-randomised control trials • Systematic or other reviews (to screen for additional original articles)	Treatment guidelines, mathematical models, editorials, viewpoints, commentaries
Timing	None	
Sector	Services to the general public provided and or managed by government health infrastructure, or through non-governmental organisations	
Required descriptive data about model	• Population/target groups • Type of patients • Community site • Health provider cadre • Frequency of service • Other services provided within the same care-model, e.g. arterial hypertension, HIV, tuberculosis	• Incomplete information that impedes full model characterization and definition

### Stage 3: Study selection

Initially, two reviewers (EF and LG) will independently screen abstracts based on the pre-defined inclusion and exclusion criteria. Studies will be classified as ‘included’ if they meet the inclusion criteria, ‘excluded’ as per the inclusion and exclusion criteria, or ‘pending’ if inclusion or exclusion cannot be immediately determined. Afterwards, full texts of all included and pending studies will be retrieved and the two independent reviewers will screen the full text for inclusion. Any disagreements during the screening process will be resolved by a meeting of the reviewers. Studies which were initially included but excluded during screening of the full text will be specifically labelled as such in a table of excluded studies including the reason for exclusion. Studies that were initially ‘pending’ but later included on closer application of criteria to full text will be documented similarly. ^19^

### Stage 4: Charting the data

A data extraction tool will be created to electronically capture relevant information from each included study. Extracted data will include information on journal, authors and dates, study design, participants, type of community-based care model, and assessed outcomes ( Table 4). The data extraction tool will be piloted on a subset of studies. Where applicable, outcomes in a comparator arm (facility-based care) will be extracted. Similar to the selection process, the extraction of data will be done in duplicate by two researchers independently, and any discrepancies will be iteratively discussed and resolved within the team.

**Table 4.** Fields to be extracted from included studies.

Parameter	Field
Publication identifiers	Authors Publication title Publication type Date of publication Journal
Study	Design Data collection dates and duration Study locations/sites
Population	Age grouping Sex
Intervention	Location of service delivery Frequency of interaction at community-site Frequency of interaction at the health care facility Cadre of healthcare provider Services provided
Outcome	Where reported: Fasting blood glucose values Random blood glucose values Glycated haemoglobin (HbA1c) values Development of complications like retinopathy, nephropathy, diabetic foot syndrome, cardiovascular diseases and cerebrovascular diseases Rates of engagement in care Acceptability to patients or providers Feasibility to implement

### Stage 5: Collating, summarising and reporting the results

A Preferred Reporting Items for Systematic reviews and Meta-Analyses extension for Scoping Reviews (PRISMA-ScR) flow diagram will be used to illustrate final numbers from included/excluded articles to fully reviewed studies. Studies will be grouped according to the type of care model and categorized according to outcomes reported. Study findings will be synthesized using narrative reporting based on themes that emerge from the extracted data. Where outcomes are stated for facility-based care, exploratory within-study comparison of outcomes will be described.

### Stage 6: Expert consultation

We will consult experts on community-based diabetes care for input. This input will help to confirm and interpret out findings, as well as contextualize implications of the findings.

## Ethics

Ethical clearance will not be required for this study as this review will utilize publicly available data.

## Data availability

### Underlying data

No underlying data are associated with this article.

### Extended data

Figshare: Community-based models of care for management of type 2 diabetes mellitus among non-pregnant adults in sub-Saharan Africa: a scoping review search strategy, https://doi.org/10.6084/m9.figshare.14610090.v3. ^21^

This project contains details of the search string in Embase.

### Reporting guidelines

Figshare: PRISMA-P checklist for “Community-based models of care for management of type 2 diabetes mellitus among non-pregnant adults in sub-Saharan Africa: a scoping review protocol”, https://doi.org/10.6084/m9.figshare.14762403.v1. ^22^

Data are available under the terms of the Creative Commons Attribution 4.0 International license (CC-BY 4.0).

## Competing interests

No competing interests were declared.
