# Supplementary material for: Community-based models of care for management of type 2 diabetes mellitus among non-pregnant adults in sub-Saharan Africa: a scoping review protocol
Source: F1000Res. 2022 Jan 18;10:535. Originally published 2021 Jul 5. [Version 2] doi: 10.12688/f1000research.52114.2 (PMC8961197; doi:10.12688/f1000research.52114.2)
Supplement: Peer review history

# Peer review: Reviewer response for version 2

**DOI:** 10.5256/f1000research.119240.r120254

**Published:** 2022-03-28

**Authors:** Anna Wahyuni Widayanti

**Affiliations:** 1 Department of Pharmaceutics, Faculty of Pharmacy, Universitas Gadjah Mada, Yogyakarta, Indonesia

The authors have responded to all comments and suggestions. No further comments. Thank you.

Is the study design appropriate for the research question?

Partly

Is the rationale for, and objectives of, the study clearly described?

Partly

Are sufficient details of the methods provided to allow replication by others?

Partly

Are the datasets clearly presented in a useable and accessible format?

Not applicable

Reviewer Expertise:

Medicine taking behavior; community-based interventions; pharmacy practice

I confirm that I have read this submission and believe that I have an appropriate level of expertise to confirm that it is of an acceptable scientific standard.

# Peer review: Reviewer response for version 2

**DOI:** 10.5256/f1000research.119240.r120253

**Published:** 2022-01-25

**Authors:** Mahmoud M. Werfalli

**Affiliations:** 1 Division of Endocrinology and Diabetic Medicine, Department of Medicine, Faculty of Health Science, University of Cape Town, Cape Town, South Africa; 2 University of Benghazi, Benghazi, Libya

Thank you very much for responding to my comments and suggestions. The manuscript is now much improved than the previous one, I have no further comments and questions. Thanks.

Is the study design appropriate for the research question?

Partly

Is the rationale for, and objectives of, the study clearly described?

Partly

Are sufficient details of the methods provided to allow replication by others?

Partly

Are the datasets clearly presented in a useable and accessible format?

Not applicable

Reviewer Expertise:

Evidence-based medicine, evidence-based health practices, epidemiology, prevention and management of diabetes and cardiovascular diseases

I confirm that I have read this submission and believe that I have an appropriate level of expertise to confirm that it is of an acceptable scientific standard.

# Peer review: Reviewer response for version 1

**DOI:** 10.5256/f1000research.55346.r97772

**Published:** 2021-11-15

**Authors:** Anna Wahyuni Widayanti

**Affiliations:** 1 Department of Pharmaceutics, Faculty of Pharmacy, Universitas Gadjah Mada, Yogyakarta, Indonesia

In the introduction, could you please explain further how the people with diabetes in SSA countries received care in general? This may include the differences in the health system, that may lead to variabilities in the care types.

 Please be clearer in stating the rationale of the study. At the beginning, the authors only explained about community-based care, but later the authors also want to investigate the performance of community-based care compared to facility-based care.

 It should be clearer what 'facility-based care' and 'community-based care' mean? As in other country settings, the community-based care for people with diabetes may include care managed by the health care facility, it may be conducted in the health care facilities or in the community, but in groups of community and involving not only health care professionals, but also community volunteers/cadres, as opposed to individual care provided by health care professionals.

 A systematic review may provide a better results in comparing effectiveness among different types of community-based care.

 Identifying relevant study: Is there any time frame set for the articles that will be included in the review?

Is the study design appropriate for the research question?

Partly

Is the rationale for, and objectives of, the study clearly described?

Partly

Are sufficient details of the methods provided to allow replication by others?

Partly

Are the datasets clearly presented in a useable and accessible format?

Not applicable

Reviewer Expertise:

Medicine taking behavior of and community-based intervention for people with type 2 diabetes

I confirm that I have read this submission and believe that I have an appropriate level of expertise to confirm that it is of an acceptable scientific standard, however I have significant reservations, as outlined above.

# Response

**Published:** 2021-12-22

**Authors:** Emmanuel Firima

**Affiliations:** Swiss Tropical and Public Health Institute, Switzerland

**In the introduction, could you please explain further how the people with diabetes in SSA countries received care in general? This may include the differences in the health system, that may lead to variabilities in the care types.**

**Author response:** ****We have addressed this and included a traditional approach to care in the revised version of the manuscript on lines 20 to 25 of the revised manuscript.

**Please be clearer in stating the rationale of the study. At the beginning, the authors only explained about community-based care, but later the authors also want to investigate the performance of community-based care compared to facility-based care.**

**Author response:** ****Even though our main interest is to present evidence on available community-based care models, we intend to evaluate performance against community-based care in studies where comparisons are made between community-based care and facility-based care. This has been clarified in the rationale. See lines 50 to 52.

**It should be clearer what 'facility-based care' and 'community-based care' mean? As in other country settings, the community-based care for people with diabetes may include care managed by the health care facility, it may be conducted in the health care facilities or in the community, but in groups of community and involving not only health care professionals, but also community volunteers/cadres, as opposed to individual care provided by health care professionals**.

**Author response:** ****Thank you for these comments. We have clarified the term ‘community-based care’ and ‘facility-based care’ in lines 26 to 30.

**A systematic review may provide a better results in comparing effectiveness among different types of community-based care.**

**Author response:** ****As this is an area with little evidence, we opted to conduct a scoping review to map out what evidence is available.

**Identifying relevant study: Is there any time frame set for the articles that will be included in the review?**

**Author response:** ****There is no restriction on the time/date the study was conducted. See lines 126 and 127 of the revised manuscript.

 Thank you.

# Peer review: Reviewer response for version 1

**DOI:** 10.5256/f1000research.55346.r90061

**Published:** 2021-08-13

**Authors:** Mahmoud M. Werfalli

**Affiliations:** 1 Division of Endocrinology and Diabetic Medicine, Department of Medicine, Faculty of Health Science, University of Cape Town, Cape Town, South Africa; 2 University of Benghazi, Benghazi, Libya

**Title**

 Are the authors focusing only on sub-Sahara Africa (SSA) or all African countries? Which classification have they used, WHO, IDF, etc.

**Introduction**

 Please elaborate further and explain why the review questions/objectives lend themselves to a scoping review approach. The authors may consider rephrasing the Introduction section to help the readers understand the contributions of this study in an explicit manner. For example, some parts of the content, such as related work, could be moved to a new related work section in particular up-to-date published related work.

 In SSA there is a wide range of community-based care for diabetes care including for non-pregnant women - for example, in South Africa and other southern countries where most of those models are operating in the community health centers (CHCs) (facility-based) via CHWs in the community. I am wondering in the search strategy, how many articles addressing the community-based models in those facilities would be missed from these communities?

**Methods**     

***Study design***

 I would suggest the review be restricted to RCTs (and possibly other controlled designs but not observational studies), which would make it more likely that a meta-analysis would be appropriate and the review might benefit from the pooling of the data.

 The authors stated: "We will conduct this scoping review using the six-stage approach initially developed by Askey and O’Malley, which has been further refined by Levac *et al.* (2010) and the Joanna Briggs Institute methods of evidence synthesis...". I would suggest to the authors using the updated framework: Munn et al. 2018 ^1^:

- As a precursor to a systematic view.
- To identify the types of available evidence in a given field.
- To identify and analyze knowledge gaps.
- To clarify key concepts/ definitions in the literature.
- To examine how research is conducted on a certain topic or field.
- To identify key characteristics or factors related to a concept.

***Identifying relevant studies***

 I would suggest using the **African search filter **for a comprehensive and strengthing search strategy.

***Participants***

 "...adults who have been diagnosed with T2DM using the standard diagnostic criteria.": Please clarify what do you mean by standard? Do the authors mean WHO diagnostic methods or using others?

***Intervention***

 "Intervention will be the delivery of care different from the traditional facility-based care model, which attempts to make care available in the community, at patients’ homes, or a central, non-formal health facility location where patients with similar conditions can access care.": This is a confusing statement. Please justify as I have mentioned the terms community and facility-based care are used interchangeably.

***Comparator***

 "With facility-based care, where available.": If not available, would it be compared to the usual care in the facility provided that facility doesn't refer to the community center, for example in South Africa?

***Outcome***

 "Of primary interest will be clinical outcomes like blood glucose indices and T2DM complications. Also of interest will be engaged in care, and acceptability of care to patients and providers.": Please categorize your outcomes as primary outcomes (clinical) and secondary. Please indicate how the authors define acceptability of care and how will be measured? 

 Thanks.

Is the study design appropriate for the research question?

Partly

Is the rationale for, and objectives of, the study clearly described?

Partly

Are sufficient details of the methods provided to allow replication by others?

Partly

Are the datasets clearly presented in a useable and accessible format?

Not applicable

Reviewer Expertise:

Evidence-based medicine, evidence-based health practices, epidemiology, prevention and management of diabetes and cardiovascular diseases

I confirm that I have read this submission and believe that I have an appropriate level of expertise to confirm that it is of an acceptable scientific standard, however I have significant reservations, as outlined above.

## References

[1] : Systematic review or scoping review? Guidance for authors when choosing between a systematic or scoping review approach. *BMC Medical Research Methodology* .2018;18(1) : 10.1186/s12874-018-0611-x 10.1186/s12874-018-0611-x 30453902PMC6245623

# Response

**Published:** 2021-12-22

**Authors:** Emmanuel Firima

**Affiliations:** Swiss Tropical and Public Health Institute, Switzerland

*Title*

**Are the authors focusing only on sub-Sahara Africa (SSA) or all African countries? Which classification have they used, WHO, IDF, etc.**

**Author response:** We focused only on sub-Saharan Africa, using geographic regions classification according to the methodology of the United Nations Statistics Division. See lines 118 and 119 of the revised manuscript.

*Introduction*

**Please elaborate further and explain why the review questions/objectives lend themselves to a scoping review approach. The authors may consider rephrasing the Introduction section to help the readers understand the contributions of this study in an explicit manner. For example, some parts of the content, such as related work, could be moved to a new related work section in particular up-to-date published related work.**

**Author response:** ****We have significantly revised the introduction section as well as the study design paragraph of the methods section to further clarify the contributions of this work and why we opted for the scoping review approach.

**In SSA there is a wide range of community-based care for diabetes care including for non-pregnant women - for example, in South Africa and other southern countries where most of those models are operating in the community health centers (CHCs) (facility-based) via CHWs in the community. I am wondering in the search strategy, how many articles addressing the community based models in those facilities would be missed from these communities?**

**Author response:** ****We understand that some community models of interest emerge or operate in health centers. Working together with a librarian, we attempted to make the search strategy very comprehensive, not excluding such community models. However, since we are interested in community models that are not simply ‘add-ons’ to facility care, in the full-text review, only such models are included.

*Methods*

*Study design*

**I would suggest the review be restricted to RCTs (and possibly other controlled designs but not observational studies), which would make it more likely that a meta-analysis would be appropriate and the review might benefit from the pooling of the data.**

**Author response:** Thank you for this comment. We anticipate very little evidence within this area. Thus, our initial interest is to map out what evidence is available to inform more research. Thus, by restricting to RCTs, we might miss out on models of community care that were possibly being implemented in observational studies.

**The authors stated: "We will conduct this scoping review using the six-stage approach initially developed by Askey and O’Malley, which has been further refined by Levac et al. (2010) and the Joanna Briggs Institute methods of evidence synthesis...". I would suggest to the authors using the updated framework: Munn et al. 2018:**

**○ As a precursor to a systematic view.**

**○ To identify the types of available evidence in a given field.**

**○ To identify and analyze knowledge gaps.**

**○ To clarify key concepts/ definitions in the literature.**

**○ To examine how research is conducted on a certain topic or field.**

**○ To identify key characteristics or factors related to a concept**.

**Author response:** This is a good suggestion. We have incorporated statements from this framework. See lines 55 and 56 of the revised manuscript. But we also kept the six-stage approach as clear steps on how we intend to implement the review.

*Identifying relevant studies*

**I would suggest using the African search filter for a comprehensive and strengthening search strategy.**

**Author response:** This has been considered by our librarians in the search. Thank you for the suggestion

*Participants*

**"...adults who have been diagnosed with T2DM using the standard diagnostic criteria.": Please clarify what do you mean by standard? Do the authors mean WHO diagnostic methods or using others?**

**Author response:** We mean the WHO diagnostic criteria. We have clarified this on lines 131 and 132 of the revised manuscript, and also added a reference. Thank you for this comment.

*Intervention*

**"Intervention will be the delivery of care different from the traditional facility-based care model, which attempts to make care available in the community, at patients’ homes, or a central, nonformal health facility location where patients with similar conditions can access care.": This is a confusing statement. Please justify as I have mentioned the terms community and facility-based care are used interchangeably.**

**Author response:** This statement has been revised to reflect our purpose. See lines 140 and 141. Also, the terms ‘community-based care’ and ‘facility-based care’ have been clarified. See lines 23 to 27.

*Comparator*

**"With facility-based care, where available.": If not available, would it be compared to the usual care in the facility provided that facility doesn't refer to the community center, for example in South Africa?**

**Author response:** If the primary study does not compare a community model to facility model, then we will consider that suitable comparison was not available.

*Outcome*

**"Of primary interest will be clinical outcomes like blood glucose indices and T2DM complications. Also of interest will be engaged in care, and acceptability of care to patients and providers.": Please categorize your outcomes as primary outcomes (clinical) and secondary. Please indicate how the authors define acceptability of care and how will be measured?**

**Author response:** The outcomes have been clearly categorized into primary and secondary. We intend to define and measure acceptability using scales adopted for each study by the authors. See lines 147 to 150 of the revised manuscript.

 Thank you.
